# Transcriptome analysis of peripheral blood from patients with rheumatoid arthritis: a systematic review

**DOI:** 10.1186/s41232-018-0078-5

**Published:** 2018-11-05

**Authors:** Shuji Sumitomo, Yasuo Nagafuchi, Yumi Tsuchida, Haruka Tsuchiya, Mineto Ota, Kazuyoshi Ishigaki, Akari Suzuki, Yuta Kochi, Keishi Fujio, Kazuhiko Yamamoto

**Affiliations:** 1 Department of Allergy and Rheumatology, Graduate School of Medicine, the University of Tokyo, 7-3-1 Hongo, Bunkyo-ku, Tokyo, 113-8655 Japan; 2 Laboratory for Statistical Analysis, Center for Integrative Medical Sciences, the Institute of Physical and Chemical Research (RIKEN), 1-7-22 Suehirocho, Tsurumi-ku, Yokohama City, Kanagawa 230-0045 Japan; 3 Laboratory for Autoimmune Diseases, Center for Integrative Medical Sciences, the Institute of Physical and Chemical Research (RIKEN), 1-7-22 Suehirocho, Tsurumi-ku, Yokohama City, Kanagawa 230-0045 Japan; 4 Center for Integrative Medical Sciences, the Institute of Physical and Chemical Research (RIKEN), 1-7-22 Suehirocho, Tsurumi-ku, Yokohama City, Kanagawa 230-0045 Japan

**Keywords:** Rheumatoid arthritis, Transcriptome analysis, RNA sequencing (RNA-seq)

## Abstract

In the era of precision medicine, transcriptome analysis of whole gene expression is an essential technology. While DNA microarray has a limited dynamic range and a problem of background hybridization, RNA sequencing (RNA-seq) has a broader dynamic range and a lower background signal that increase the sensitivity and reproducibility. While transcriptome analyses in rheumatoid arthritis (RA) have generally focused on whole peripheral blood mononuclear cells (PBMC), analyses of detailed cell subsets have an increased need for understanding the pathophysiology of disease because the involvement of CD4^+^ T cells in the pathogenesis of RA has been established. Transcriptome analysis of detailed CD4^+^ T cell subsets or neutrophils shed new light on the pathophysiology of RA. There are several analyses about the effect of biological treatment. Many studies report the association between type I interferon signature gene expression and response to therapy.

## Background

Rheumatoid arthritis (RA) is an autoimmune disease characterized by chronic inflammatory synovitis and progressive disability. Environmental factors including smoking [1] and periodontitis, epigenetic modification, and susceptibility genes [2] generate self-proteins with a citrulline residue by post-translational modification. Breakdown of immunological tolerance to citrullinated self-proteins is considered to be an important feature of RA pathogenesis [3]. This anticitrulline response can be detected in T cell and B cell compartments and is probably initiated in secondary lymphoid tissues or bone marrow. Thereafter, synovitis occurs when leukocytes infiltrate the synovial compartment [4]. Synovium is the principal target of inflammation in RA, and the resident fibroblast-like synoviocytes (FLSs) are implicated in the pathogenesis of synovitis [5].

There is much interest in transcriptome analysis as a mechanism for predicting RA pathogenesis and effect of treatment. Gene expression profiling is a quantitative measurement of messenger RNA levels for thousands of genes at once, to create a global picture of cellular function and contribute to the era of stratified medicine [6]. DNA microarrays have been used to drive genetic analyses for more than two decades. However, microarrays have limited dynamic range that often prevents accurate assessment of low signal intensities, and suffer from the problem of background hybridization. Such limitations are largely absent in RNA sequencing (RNA-seq), a next-generation sequencing (NGS) method largely used for the genome-wide measurement of RNA abundance. Compared with microarrays, RNA-seq has several advantages, such as low background signal, a broader dynamic range, increased sensitivity, and high reproducibility [7].

Transcriptome analyses in RA generally focused on whole peripheral blood mononuclear cells (PBMC) [8, 9]. However, the importance of cell-type specificity is becoming increasingly apparent in gene expression work. Cell-type specificity would be missed in whole blood samples, especially when investigating low abundance genes. Moreover, dynamic variations in leukocyte subsets may confound the interpretation of results [6].

Transcriptome analyses also contribute to the understandings of the effect of treatment. For example, transcriptome analysis revealed that type I interferon (IFN) signature genes were particularly associated with clinical response to bDMARDs including TNF-α inhibitor [10], tocilizumab [8], and rituximab [9]. In the analysis of the effect of treatment, samples must be taken at the same time points and from the same source in order to lead biologically meaningful comparative transcriptome analyses. Moreover, longitudinal studies may yield more relevant answers compared to cross-sectional studies.

In this review, we discuss the transcriptome analysis of various cell subsets in PBMC of RA and analysis about the effect of biological treatment.

## Main text

### CD4^+^ T cells

The involvement of CD4^+^ T cells in the pathogenesis of RA has been established based on the fact that HLA-DRB1 genotype is by far the strongest genetic risk factor for RA [2]. Moreover, RA genetic risk loci preferentially map to enhancers and promoters active in CD4^+^ T cell subpopulations [11]. We conducted expression quantitative trait loci (eQTL) analysis on five subsets of immune cells (CD4^**+**^ T cells, CD8^**+**^ T cells, B cells, natural killer (NK) cells, and monocytes) and unfractionated peripheral blood from 105 healthy Japanese volunteers. We developed a three-step analytical pipeline comprising (i) prediction of individual gene expression using our eQTL database and public epigenomic data, (ii) gene-level association analysis, and (iii) prediction of cell-specific pathway activity by integrating the direction of eQTL effects. By applying this pipeline to RA GWAS data sets, the tumor necrosis factor (TNF) pathway was predicted to be significantly activated, specifically in CD4^+^ T cells [12].

Ye et al. examined the whole-genome transcription profile of CD4^+^ T cells in RA compared with healthy individuals using microarray analysis [13]. They reported that CD4^+^ T cells from patients with RA have abnormal functional networks in STAT3 signaling and Wnt signaling. Their results also suggested that the aberrant expression of several zinc finger transcription factors (ZEB1, ZNF292, and ZNF644) may be potential pathogenic factors for RA.

#### CD4^+^ T cell subsets

There are few reports for detailed transcriptome analysis of CD4^+^ T cell subsets in RA. Although contributions of Th17 cells [14], regulatory T cells (Treg) [15], and follicular helper T cells (Tfh) [16] have been reported, the actual modifications of CD4^+^ T cells in RA have not been elucidated. We performed RNA-seq of seven CD4^+^ T cell subsets (naive, Th1, Th17, Th1/17, nonTh1/17, Tfh, and Treg) of peripheral blood taken from RA patients and healthy controls (HC) [17]). We found that several pathways including GTPase-associated signaling and apoptosis signaling were upregulated in RA as previously reported [18, 19]. Weighted gene co-expression network analysis (WGCNA) [20] identified a gene module that consisted of 227 genes and was correlated with DAS28-CRP. The most highly connected 30 genes of this module included ZAP70 and JAK3, and pathway analysis of this module revealed dysregulation of the TCR signaling pathway network.

#### Co-stimulatory molecules

Abatacept is a fusion protein composed of the Fc region of immunoglobulin and the extracellular domain of CTLA-4 that inhibits T cell activation by blocking co-stimulation [21]. We performed RNA-seq of seven CD4^+^ T cell subsets (naive, Th1, Th17, Th1/17, nonTh1/17, Tfh, and Treg) of peripheral blood taken from RA patients before and 6 months after abatacept treatment [17]. Overview of expression pattern of RA revealed that administration of abatacept exerts a large shift toward the expression pattern of healthy control. Knowledge-based pathway analysis revealed the upregulation of activation-related pathways in RA that was substantially ameliorated by abatacept. We found that dysregulated TCR signaling pathways in RA were detected in RA through CD4^+^ T cell subsets and ameliorated by abatacept treatment.

### B cells

Rituximab is monoclonal anti-CD20 antibody and used to treat RA and B cell non-Hodgkin’s lymphoma although RA treatment has not been approved in Japan. Sellam et al. performed microarray of whole peripheral blood samples of RA patients before and 24 weeks after treatment of rituximab [9]. They detected signature 143 genes for response featured upregulation of inflammatory genes centered on NF-kB, including IL33 and STAT5A, and downregulation of the IFN pathway. This signature accurately identifies patients with RA who will not respond to rituximab therapy. Raterman et al. also performed microarray of whole peripheral blood samples and found that the baseline expression of type I IFN response genes (LY6E, HERC5, IFI44L, ISG15, MxA, MxB, EPSTI1, and RSAD2) could be useful as a predictive biomarker for non-response to rituximab in RA [22]. Relationship between the type I IFN signature and the response to rituximab in RA had been reported by others [23, 24].

### Neutrophils

Wright et al. performed RNA-Seq of RA neutrophils to identify pre-therapy gene expression signatures that correlate with disease activity or response to TNF inhibitor (TNFi) therapy [10]. Pathway analysis predicted activation of IFN signaling in RA neutrophils, identifying 178 IFN-response genes regulated by IFN-α, IFN-β, or IFN-γ. Patients could be categorized as IFN-high or IFN-low. Patients in the IFN-high group achieved a better response to TNFi therapy than patients in the IFN-low group. IFN-response genes are significantly upregulated in RA neutrophils compared with healthy controls. Higher IFN-response gene expression in RA neutrophils correlates with a good response to TNFi therapy.

### Proinflammatory cytokines

Koczan et al. performed microarray of PBMC of RA patients before the first application of the TNFα blocker etanercept as well as after 72 h [25]. Early downregulation of expression levels was associated with good clinical responses. Informative gene sets include genes (NFKBIA, CCL4, IL8, IL1B, TNFAIP3, PDE4B, PPP1R15A, and ADM) involved in different pathways and cellular processes such as TNFα signaling via NFκB; Van Baarsen et al. performed microarray of PBMC of RA patients before and 1 month after infliximab treatment [26]. While the change in IFN response genes was unrelated to baseline expression levels, treatment-induced increase of IFN response gene activity was associated with poor clinical response to infliximab treatment.

Sanayama et al. performed genome-wide DNA microarray of PBMC of RA patients before and after treatment with an anti-IL-6 receptor antibody, tocilizumab [8]. They found that three type I IFN response genes (IFI6, MX2, and OASL) and MT1G was significantly different between nonresponders and responders. MT1G encodes metallothionein-1G, a member of the metallothionein (MT) proteins that are involved in protection against oxidative stress and inflammatory responses. The MT-1 promoter contains a STAT binding site, and the gene expression of MT-1 is directly upregulated by IL-6. They suggested that type I IFN signaling and metallothioneins are involved in the pathophysiology of RA. Saito et al. performed microarray of CD4^+^ T cells before and after treatment with tocilizumab [27]. They found that ARID-5A was downregulated by tocilizumab therapy. ARID-5A was a lineage-specific attenuator of Th17 cell differentiation and might be involved in the pathogenesis of RA.

### Future perspective

While we have reviewed transcriptome analysis of peripheral blood from RA patients, there are several reports of specimens other than peripheral blood, mainly synovial tissue [28] and FLS [29]. Dennis et al. revealed that baseline synovial myeloid, but not lymphoid, gene signature expression was higher in patients with good compared with poor clinical response to anti-TNFα therapy [28]. Galligans et al. analyzed gene expression of FLS obtained from RA and osteoarthritis (OA), and identified 34 genes specific to RA and OA FLS, and 8 genes correlated with RA disease activity. Epigenetic evaluation of FLS has also been extensively studied [30], and recently, Ai et al. reported comprehensive epigenetic landscape of RA FLS including histone modifications (H3K27ac, H3K4me1, H3K4me3, H3K36me3, H3K27me3, and H3K9me3), open chromatin, RNA expression, and whole-genome DNA methylation [31].

There are several reports concerning the effect of treatments other than biologic treatment on gene expression. Blits et al. investigated peripheral blood cells from methotrexate (MTX)-naïve and MTX-treated RA patients as well as from healthy controls. Concurrent with an immune activation gene signature, a significant upregulation of folate metabolizing enzymes (g-glutamyl hydrolase, dihydrofolate reductase), and MTX/folate efflux transporters (ABCC2 and ABCC5) was observed in MTX-naïve RA patients. Strikingly, MTX treatment normalized such differential gene expression levels to those observed in healthy controls [32]. Moreover, there is a report about effect of JAK inhibitor tofacitinib on gene expression of RA FLS [33]. Tofacitinib reduces matrix metalloproteinase (MMP)-1 and MMP-3 and IFN-regulated chemokines CCL2, CXCL10, and CXCL13 expression.

Type I IFNs are cytokines that regulate antiviral immune responses. Upregulation of type I IFN signature genes in RA [8, 10], and its relationship with therapeutic reactivity [8, 10, 23, 24] has been reported. The relationship between the type I IFN signature and the humoral autoimmune response in RA was analyzed in a number of previous studies. The presence of an IFN signature was associated with the persistence of ACPA after TNF blockade [26]. It has been hypothesized that patients with high activity of type I IFN may respond better to TNF blockade because of the anti-inflammatory effects of their disease-associated high levels of IFNβ. Alternatively, patients with an IFN high signature may have an overall higher level of inflammatory activity than do patients with an IFN low signature and may respond better to TNF blockade because of the higher TNFα activity.

Okada et al. described that comprehensive genetic study sheds light on fundamental genes, pathways, and cell types that contribute to RA pathogenesis and provides important information for drug discovery [2]. On the other hand, merely gene expression analysis has a limitation for precision treatment for RA, for example, upregulation of type I IFN signature gene were detected commonly in autoimmune disease including RA, systemic lupus erythematosus [34], dermatomyositis [35], and systemic sclerosis [36]. It is necessary to increase the number of patients for analysis and to integrate more precise clinical information, genomic and epigenetic data, and gene expression data of various cell subsets. Recently, data of 1424 early RA patients from two consortia were combined to carry out a genome-wide study of response to MTX. The strongest evidence for association was with rs168201 in NRG3, and some support was also seen for association with ZMIZ1, previously highlighted in a study of response to MTX in juvenile idiopathic arthritis [37].

## Conclusion

In the era of precision medicine, transcriptome analysis of whole gene expression is an essential technology. Analyses of detailed cell subsets should have an increased need for understanding the pathophysiology of disease. Careful and extensive consideration of study design is necessary for successful gene expression studies because gene expression is dependent on stages of the disease, time course of treatment, type of tissue, and cell types. While testing peripheral blood has been a standard transcriptome analysis of RA patients, it would be better to test synovial tissue, as this is the site of inflammation. The disadvantage of synovial tissue approach is that arthroplasty and blind needle biopsy had not been widely available, although the use of arthroscopic and ultrasonographic technologies has improved the reliability of synovial biopsies. Researchers will need to carefully consider the advantages of using peripheral blood samples to investigate.

## Abbreviations

DAS28-CRP: Disease activity score- CRP
eQTL: Expression quantitative trait loci
FLS: Fibroblast-like synoviocyte
GWAS: Genome-wide association study
IFN: Interferon
MTX: Methotrexate
NGS: Next-generation sequencing
OA: Osteoarthritis
PBMC: Peripheral blood mononuclear cell
RA: Rheumatoid arthritis
RNA-seq: RNA sequencing
TCR: T cell receptor
Tfh: Follicular helper T cells
TNF: Tumor necrosis factor
Treg: Regulatory T cells
WGCNA: Weighted gene co-expression network analysis
